# Evaluation of breast lesions using a newly developed 3D breast ultrasound imaging device: a preliminary study on efficacy and validity

**DOI:** 10.1038/s41598-026-48519-x

**Published:** 2026-04-27

**Authors:** Miribi Rho, Kyunghwa Han, Min Jung Kim

**Affiliations:** 1 Department of Radiology, Severance Hospital, Research Institute of Radiological Science, Center for Clinical Imaging Data Science, Yonsei University College of Medicine, 50-1 Yonsei-ro Seodaemun-gu, Seoul, 03722 South Korea; 2 Department of Integrative Medicine Major in Digital Healthcare, Yonsei University College of Medicine, Seoul, 06273 South Korea

**Keywords:** Automated breast ultrasound, BI-RADS, Breast neoplasms, Ultrasonography, Image quality, Cancer, Diseases, Medical research, Oncology

## Abstract

To evaluate the image quality and diagnostic performance of a newly developed three-dimensional automated breast ultrasound (ABUS) system, MammouS-N, characterized by mammography-like geometry. In this prospective study of 121 women with 139 breast lesions, MammouS-N images were obtained in three different projections and reviewed in consensus by two breast radiologists blinded to prior imaging and pathology. Image quality was rated for six parameters. Lesion detectability and BI-RADS categories were compared between MammouS-N and hand-held ultrasound (HHUS), and their concordance was assessed using κ statistics. Diagnostic performance was assessed using the receiver operating characteristic (ROC) analysis with pathology as the reference standard. MammouS-N achieved diagnostically acceptable image quality (score ≥ 4) in over 90% of cases and near-complete coverage in 98.6% of breasts. Of 139 lesions, 131 (94.2%) were visualized, including 88 of 92 malignant lesions (95.7%). The area under the ROC curve for malignancy detection was 0.893 for MammouS-N (95% CI, 0.844–0.943) and 0.923 (95% CI, 0.885–0.962) for HHUS, without a significant difference (*p* = 0.207). BI-RADS concordance between MammouS-N and HHUS showed moderate-to-substantial agreement (Cohen’s κ = 0.45; weighted κ = 0.65), with a mild benign-leaning tendency, as downgrades exceeded upgrades (2.1% of benign and 12.0% of malignant lesions were downgraded relative to HHUS. MammouS-N demonstrated high image quality, excellent lesion detectability, and comparable diagnostic concordance with HHUS. Its mammography-aligned geometry facilitates spatial correspondence across modalities, supporting its potential as a complementary imaging tool for breast cancer screening.

**Supplementary Information:**

The online version contains supplementary material available at 10.1038/s41598-026-48519-x.

## Introduction

Breast cancer remains one of the most common malignancies among women worldwide and a leading cause of cancer-related mortality^1^. The prognosis of breast cancer is strongly dependent on early detection. Patients diagnosed at an early stage generally show significantly higher survival rates, whereas those diagnosed at advanced stages exhibit poorer outcomes. Mammography has long served as the standard screening modality, and population-based screening programs have been shown to reduce breast cancer-specific mortality by approximately 20%^2^. Nevertheless, dense breast tissue reduces the diagnostic sensitivity of mammography^3^.

High breast density not only obscures lesions on mammograms but also acts as an independent risk factor for breast cancer^4^. Consequently, supplemental imaging modalities have been introduced to improve diagnostic accuracy in women with dense breasts. Among these, ultrasonography is the most widely used adjunct technique^5^. However, conventional hand-held ultrasound (HHUS) has several inherent limitations, including operator dependency and variability in image acquisition. Furthermore, correlating ultrasonographic findings with mammographic images which are acquired across different planes demands substantial expertise^6^. To address these limitations, automated breast ultrasound (ABUS) has been developed to reduce operator dependency and standardize image acquisition, thereby improving diagnostic consistency^7,8^. Nevertheless, existing ABUS systems differ from mammography in imaging orientation, which limits direct spatial concordance and complicates multimodal image interpretation.

Recently, several studies proposed hybrid imaging systems that integrate mammographic acquisition geometry with ultrasound technology^9,10^. These systems aimed to implement three-dimensional ultrasonography based on mammographic imaging geometry to facilitate anatomical spatial concordance between the two modalities and potentially improve lesion detectability. In response to this unmet clinical need, a novel three-dimensional breast ultrasound imaging device - the *MammouS-N* (MedicalPark, Gyeonggi, Korea) - was developed. The system employs dual high-frequency linear transducers, to scan a wide field of view (FOV) and acquire volumetric breast data. This design enables comprehensive visualization of breast anatomy and precise evaluation of lesions within a standardized imaging plane comparable to mammography.

This preliminary study aims to evaluate the image quality of the *MammouS-N* system and the clinical feasibility of its application in the detection and assessment of breast lesions.

## Methods

This prospective, single-center observational study was approved by the Institutional Review Board (IRB) of Severance Hospital, Yonsei University College of Medicine (IRB No. 2023-2172-006). All participants provided written informed consent after receiving a detailed explanation of the study protocol and objectives, in accordance with the Declaration of Helsinki.

### Study population

Between October 2023 and March 2024, women aged 20 years or older who presented with breast lesions requiring biopsy, or who had undergone tissue sampling within the previous two years with confirmed pathological results, were prospectively enrolled.

Exclusion criteria were as follows: (1) prior breast surgery or breast implant insertion; (2) failure to establish histopathologic diagnosis; and (3) refusal of study participation.

A total of 139 biopsy proven breast lesions of 121 eligible patients were evaluated using the MammouS-N system and included in the final analysis as clinically relevant lesions. Multiple lesions per patient were possible, including unilateral or bilateral involvement. Breast composition was assessed on mammography according to the American College of Radiology Breast Imaging Reporting and Data System (BI-RADS)^11^: 11 women (9.1%) had category B breasts, 80 (66.1%) category C, and 25 (20.7%) category D. Mammographic breast density was not available for five women (4.1%) who did not undergo mammography due to young age.

### Equipment/imaging protocol

All participants underwent standard-of-care digital mammography and hand-held ultrasound as part of their clinical evaluation, followed by a three-dimensional automated breast ultrasound (MammouS-N) examination prior to biopsy.

The MammouS-N system (MedicalPark, Gyeonggi, Korea) is an automated three-dimensional breast ultrasound device designed to mimic mammographic imaging geometry. It utilizes dual high-frequency linear probes (5–12 MHz) with 1024 elements each and a wide FOV (220 × 300 mm), enabling high-resolution volumetric scanning of the entire breast.

**Fig. 1:**
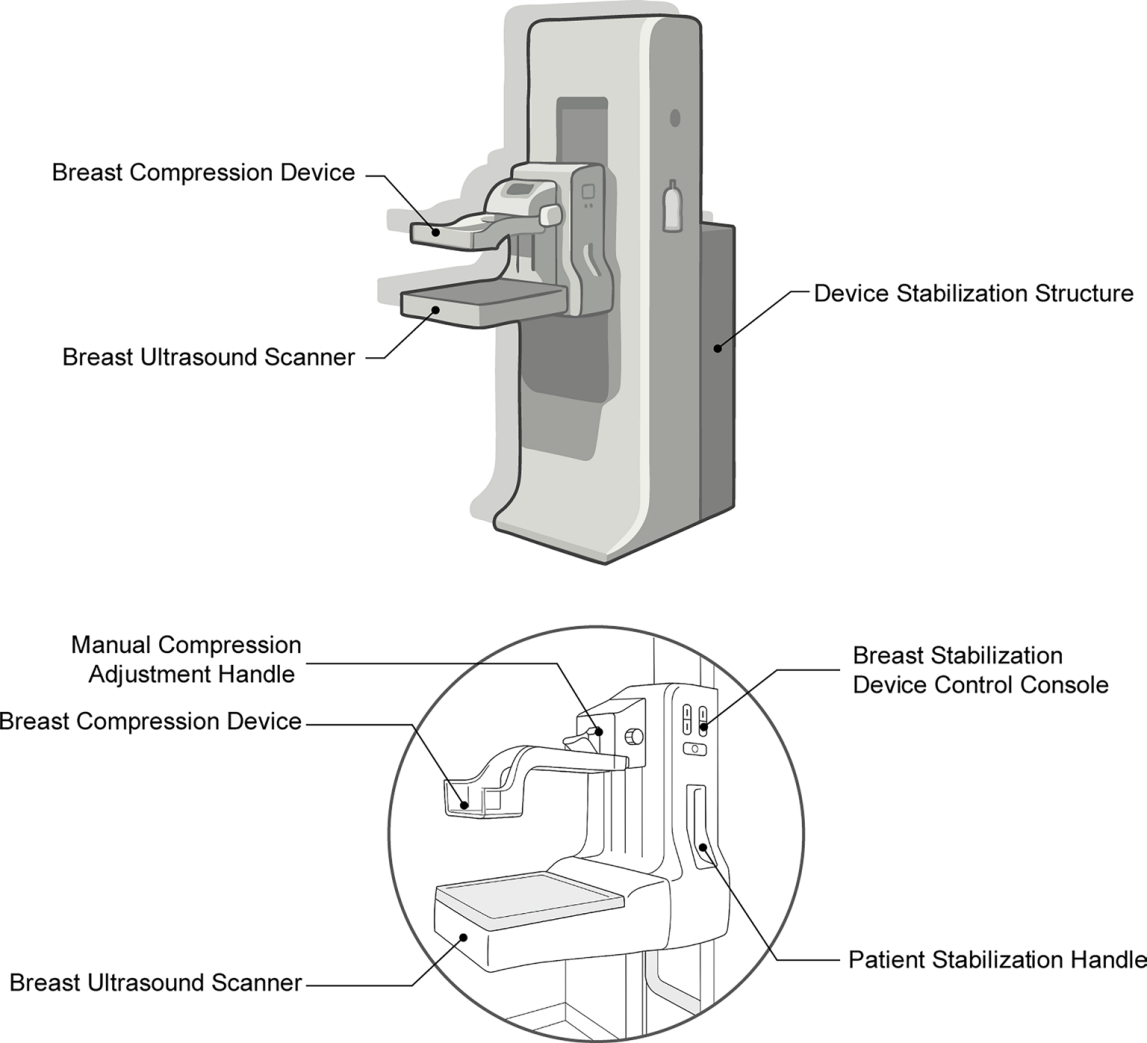
Structural components and dual transducer configuration of the MammouS-N automated breast ultrasound device.

**Fig. 2:**
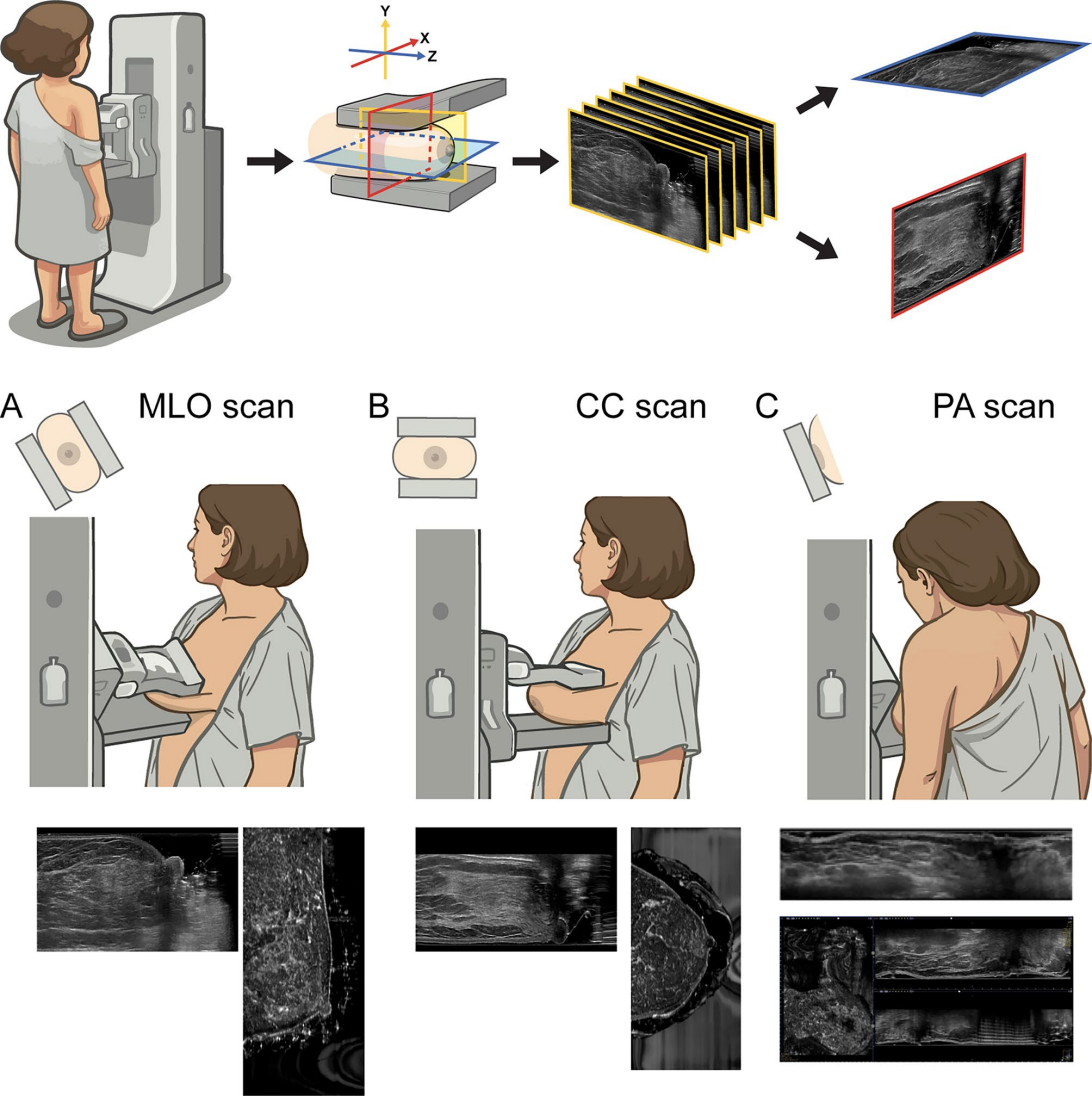
Schematic illustration of MammouS-N image acquisition in standard breast views. The breast is positioned between the ultrasound scanner and the compression device in a manner analogous to conventional mammography. During scanning, the transducer traverses the compressed breast while continuously acquiring transverse ultrasound images. These transverse images are subsequently reconstructed into three-dimensional volumetric datasets, from which view-specific images corresponding to mammographic standard acquisition planes. Imaging is sequentially performed in three standard views: (A) mediolateral oblique (MLO), (B) craniocaudal (CC), and (C) posteroanterior (PA). These schematic representations illustrate the orientation of the breast during MammouS-N scanning.

The breast was positioned between the transducer and compression panel in a manner analogous to mammography. Imaging was sequentially obtained in three standard views: mediolateral oblique (MLO), craniocaudal (CC), and posteroanterior (PA) (Figs. 1 and 2) by two radiologic technologists, each with more than ten years of mammography experience. For the PA view, the breast was gently compressed directly against the transducer surface without panel compression, allowing the acquisition of anterior breast structures. The same acquisition sequence was repeated for the contralateral breast.

### Image review

All MammouS-N images were reviewed in consensus by two experienced breast radiologists who were blinded to prior imaging and pathology results. The primary endpoints of this study were image quality and lesion detectability. Image analysis was performed in sequential order, beginning with the assessment of image quality based on normal breast tissue on MammouS-N images, followed by lesion detection, assessment of imaging quality of detected lesions, and their classification according to the BI-RADS^11^.

### Image quality

Image quality was evaluated across six key parameters: axial resolution, anatomic differentiation, tissue contrast, lesion conspicuity, artifact and coverage adequacy.

As for axial resolution, anatomic differentiation, tissue contrast and lesion conspicuity, each parameter was graded on a five-point ordinal scale (1 = non-acceptable, 2 = inadequate, 3 = inferior, 4 = comparable, 5 = equal to HHUS) to enable a direct comparison with standard HHUS images. Scores of 1–2 were considered non-diagnostic, indicating complete or near-complete loss of structural delineation (score 1) and insufficient for diagnostic interpretation (score 2). Scores of 3–5 were regarded as follows: denoted image quality inferior to HHUS (score 3), comparable quality (score 4), and equal or superior image quality (score 5).

Artifacts were evaluated using a five-point scale, focusing on acoustic distortions caused by nipple shadowing, air bubbles, or skin separation. Scores were defined as follows: 1 = severe artifact-obscuring lesions or anatomical structures; 2 = moderate posterior shadowing partially masking the lesion; 3 = mild shadowing without diagnostic interference; 4 = minimal shadowing with incomplete nipple visualization; and 5 = no artifacts with clearly visualized nipple and posterior margin. Coverage adequacy was evaluated for each imaging plane—mediolateral oblique (MLO), craniocaudal (CC), and posteroanterior (PA). For the MLO and CC views, the extent of breast inclusion was compared with the corresponding mammographic projections to verify complete coverage of the breast volume. For the PA view, adequacy was determined by confirming inclusion of the pectoralis muscle and the entire breast parenchyma within the three-dimensional ABUS dataset. All parameters were graded on a five-point scale (1 = inadequate, 2 = limited, 3 = partial, 4 = near-complete, 5 = complete coverage).

### Lesion detection and concordance analysis

Each lesion detected on *MammouS-N* images was assigned a BI-RADS category without knowledge of pathology and the mammography and HHUS results. For patients with bilateral lesions, each breast was evaluated separately, and in cases with multiple lesions within the same breast, the most suspicious lesion was selected for classification and subsequent analysis. The clinicopathologic findings were reviewed and spatial concordance was determined.

Interpretation concordance between lesions detected on MammouS-N and those identified on HHUS or confirmed histopathologically was evaluated to determine intermodality alignment and lesion-level correspondence. Each MammouS-N–detected lesion was matched with its HHUS counterpart based on anatomic location, size, morphology, and echotexture. BI-RADS categories on HHUS were based on the prospective records obtained prior to biopsy. To assess diagnostic consistency, BI-RADS categories assigned to MammouS-N images were compared with pre-biopsy BI-RADS classifications based on HHUS images.

### Factors influencing lesion detectability

Potential determinants of lesion detectability were analyzed with respect to both image quality and mammographic factors.

The effects of axial resolution, anatomic differentiation, tissue contrast, lesion conspicuity and artifacts on lesion detection were evaluated using the structured image-quality scoring described above. To assess the quantitative relationship between image quality and detectability, lesions were stratified according to their image-quality scores: high-quality (score ≥ 4) versus suboptimal (score < 4). Detection rates were then compared between these two groups to determine whether lower image quality or incomplete scans were associated with decreased lesion detectability.

Mammographic findings were reviewed to characterize background parenchymal features and lesion visibility. Breast density was graded according to the American College of Radiology (ACR) BI-RADS breast composition categories (A-D). Each lesion with a corresponding mammographic finding was categorized into one of three types based on its imaging appearance: (1) mass-type findings, including masses, focal asymmetries, or asymmetries (without associated calcifications); (2) calcification-associated lesions and (3) mammographically undetectable (not visible) lesions, defined as those not identified on mammography. These mammographic categories were subsequently used to analyze the lesion detectability of MammouS-N according to the underlying mammographic presentation.

### Statistical analysis

Continuous variables were summarized as means ± standard deviations, and categorical variables as frequencies and percentages. Image quality parameters - including axial resolution, anatomic differentiation, tissue contrast, lesion conspicuity, artifact, and coverage adequacy -along with detection rate and BI-RADS assessments, were analyzed to evaluate image acceptability and overall diagnostic performance. For bilateral or multiple lesions, the most suspicious lesion in each breast was selected and analyzed.

Interpretation concordance between BI-RADS assessments based on MammouS-N and HHUS was evaluated using Cohen’s κ and weighted κ statistics. The degree of concordance was interpreted according to conventional thresholds: κ = 0.41–0.60 indicating moderate, 0.61–0.80 substantial, and > 0.80 almost perfect agreement. Ordinal correlation between BI-RADS categories was further analyzed using the Mantel–Haenszel chi-square test. Receiver operating characteristic (ROC) curves were generated for each modality, and the area under the curve (AUC) values were compared using DeLong’s test. BI-RADS thresholds (≥ 4 A considered positive for malignancy) were used for the receiver operating characteristic (ROC) curve analysis using histopathology as the reference standard. For bilateral or multiple-lesion analyses, generalized estimating equations (GEE) were applied to adjust for intra-patient correlation. To evaluate diagnostic interpretation concordance trends, net reclassification rates were calculated to assess whether MammouS-N tended to upgrade or downgrade BI-RADS categories relative to HHUS when compared with final pathology. The reclassification rate was defined as the difference between the proportions of upgraded and downgraded BI-RADS categories from MammouS-N relative to HHUS. For malignant lesions, the rate was calculated as (upgrade − downgrade) / total malignant lesions, and for benign lesions, as (downgrade − upgrade) / total benign lesions.

Relationships between image quality parameters (axial resolution, tissue contrast, artefact, and coverage adequacy) and lesion detectability were examined using chi-square or Fisher’s exact tests, as appropriate. A binomial proportion test was also applied to determine whether diagnostically acceptable image quality (score ≥ 4) occurred in more than half of all examined cases. Finally, the influence of mammographic appearance on MammouS-N detectability was analyzed using the chi-square test. Lesion detection rates were compared across three mammographic categories—mass-type findings, calcification-associated lesions, and mammographically occult lesions—to assess potential differences according to imaging phenotype.

All statistical analyses were performed using SPSS software (version 26.0; IBM Corp., Armonk, NY, USA) and R software (version 4.5.1; R Foundation for Statistical Computing, Vienna, Austria). A *p*-value of < 0.05 was considered statistically significant.

## Results

### Patient characteristics

A total 121 patients (mean age, 45.6 ± 10.8 years; range, 28–76 years) were included in this single-center prospective study. Among them, 18 patients had bilateral breast lesions, which were evaluated separately, resulting in a total of 139 lesion analyses. Of all lesions, 92 (66.2%) lesions were histopathologically confirmed as malignant, and 47 (33.8%) were benign. Mean age was significantly higher for patients with malignant lesions (48.1 ± 11.0 years) than for those with benign lesions (40.6 ± 8.6 years). Mean lesion size was 20.7 ± 12.9 mm, with malignant lesions (23.8 ± 13.6 mm) being significantly larger than benign ones (14.6 ± 8.6 mm, *p* = 0.03). Malignant cases included invasive ductal carcinoma (*n* = 68), ductal carcinoma in situ (*n* = 16), invasive lobular carcinoma (*n* = 4), mucinous carcinoma (*n* = 3), and papillary carcinoma (*n* = 1). Benign lesions comprised fibroadenoma (*n* = 12), fibroadenomatoid hyperplasia (*n* = 3), fibrocystic change (*n* = 3), and papillary lesions (*n* = 2) and other minor histologic subtypes (Table 1).

**Table 1:** The pathology and clinical characteristics of breast lesions (139).

	N (%)
Malignancy	92 (66.2)
Invasive ductal carcinoma	68 (48.9)
Invasive lobular carcinoma	4 (2.9)
Mucinous carcinoma	3 (2.2)
Papillary carcinoma	1 (0.7)
DCIS	16 (11.5)
Benign	47 (33.8)
Fibroadenoma	17 (12.2)
Fibroadenomatoid hyperplasia	12 (8.6)
Fibrocysic change	3 (2.2)
Papillary lesion	3 (2.2)
Radial sclerosing lesion	2 (1.4)
Mucocele-like lesion	2 (1.4)
Others*	8 (5.6)

* includes lobular carcinoma in situ, adenosis, columnar cell lesion, sclerosing adenosis with apocrine metaplasia, cystic apocrine metaplasia, ectatic duct with peridutal lymphocytic infiltration, pseudoangiomatous stromal hyperplasia, stromal fibrosis.

### Image quality assessment

The MammouS-N system produced overall image quality that was comparable or equal to HHUS across most evaluation parameters (Table 2).

**Table 2:** Image quality assessment compared to hand-held ultrasound (HHUS).

	Resolution	Anatomic differentiation	Tissue contrast*	Lesion conspicuity*	Artifact
5-equal	89 (64.0)	98 (70.5)	94 (71.8)	98 (74.8)	16 (11.5)
4-comparable	42 (30.2)	32 (23.0)	28 (21.4)	24 (18.3)	94 (67.6)
3-inferior	8 (5.8)	9 (6.5)	8 (6.1)	8 (6.1)	23 (16.5)
2-inadequate	0 (0)	0 (0)	1 (0.8)	1 (0.8)	6 (4.3)
1-non acceptable	0 (0)	0 (0)	0 (0)	0 (0)	0 (0)
Total	139	139	131	131	139

* 131 lesions except for 8 non-identified lesions were analyzed; two of them is subtle on ultrasound and the others are too far lower to be included in the scan range.

Scores of ≥ 4 (“comparable” or “equal”) were achieved in 94.2% (131/139) of lesions for axial resolution, 93.5% (130/139) for anatomic differentiation, 93.1% (122/131) for tissue contrast, and 93.1% (122/131) for lesion conspicuity. Regarding tissue contrast and lesion conspicuity, 131 lesions were analyzed. Eight lesions (four malignant and four benign) were excluded because they were not clearly visualized on the MammouS-N images. Among them, two appeared only subtly on hand-held ultrasound, while the remaining four were located too deeply or peripherally to be included within the scan range. In contrast, artifact evaluation showed that 110 of 139 (76.9%) images were rated as comparable or equal to HHUS, scoring ≥ 4. although a few cases exhibited relatively lower quality due to artifacts. These artifacts were presumed to be related to inherent technical limitations during image acquisition, such as air bubbles within the coupling gel prior to ultrasound propagation through the breast tissue.

Coverage adequacy was rated complete (score = 5) in 132 examinations (95.0%). However, eight MLO views and nine CC views of the 139 total examinations were unavailable for analysis as certain images were not appropriately acquired. (Near-)complete breast coverage (score 4 or 5) was achieved in 128 of 131 (97.7%) MLO views, 100 of 130 (76.9%) CC views, and 134 of 138 (97.1%) PA views. When considering any projection, 137 of 139 (98.6%) breasts demonstrated at least one view with (near-) complete coverage (score 4 or 5) (Supplemental Table e1).

### Lesion detection and spatial concordance

**Fig. 3:**
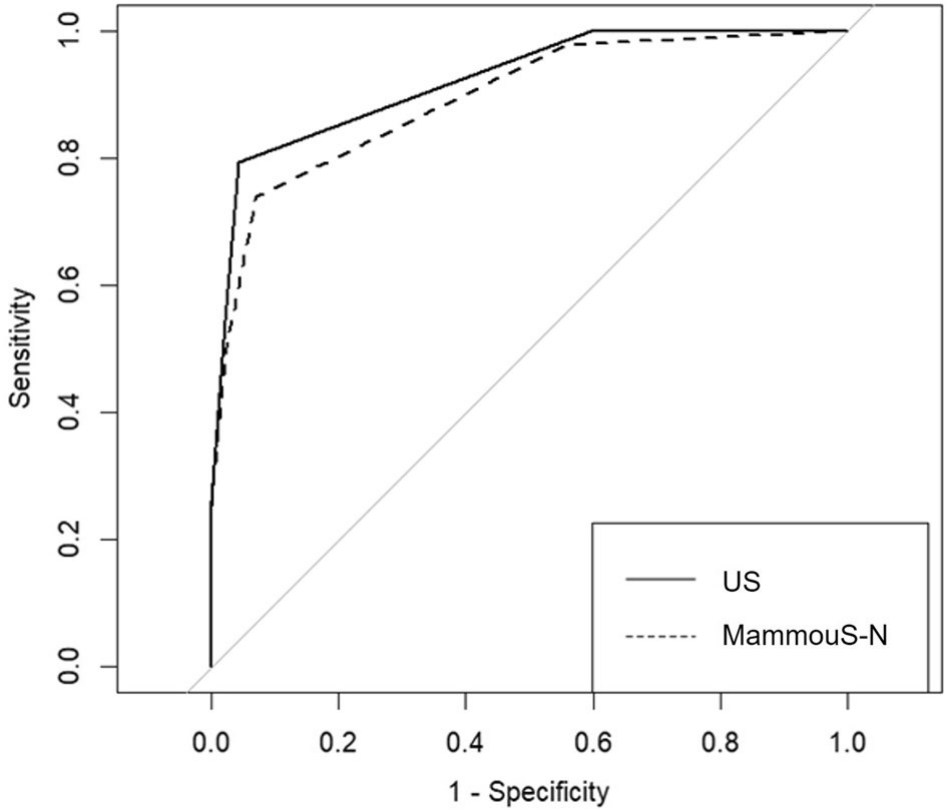
Receiver operating characteristic (ROC) curve analysis comparing MammouS-N and Hand-held ultrasound.

Of the 139 lesions, 131 (94.2%) were visualized on MammouS-N. The distribution of BI-RADS categories for these MammouS-N lesions was as follows: category 3 (21/131, 16.0%), category 4 A (42/131, 32.1%), category 4B (23/131, 17.6%), category 4 C (23/131, 17.6%), and category 5 (22/131, 16.8%). Among the 92 malignant lesions, 86 (93.5%) were classified as BI-RADS ≥ 4 on MammouS-N, while 2 (2.2%) were assigned BI-RADS 3 and later confirmed as malignant on pathology. Therefore, the detection rate among malignant lesions was 95.7% (88 of 92). Among the 47 benign lesions, 43 lesions (91.5%) were identified and 23 (48.9%) were further categorized as BI-RADS ≤ 3. Among 4 non-identified malignant lesions, two lesions were associated with microcalcifications that appeared too subtle for ultrasound visualization, and two were located in far upper medial or peripheral regions beyond the effective scanning range. The interpretation concordance between the BI-RADS assessments based on either MammouS-N and HHUS was moderate to substantial (Cohen’s κ = 0.453, weighted κ = 0.647) and strong ordinal association was noted (*p* < 0.001, Mantel–Haenszel χ² test); 71.9% of lesions (100 out of 139) were categorized in the same BI-RADS category. Specifically, 54.0% (73 of 139 lesions) remained in the same BI-RADS category even when subcategories were considered. In the reclassification analysis stratified by pathologic outcome (Table 3), MammouS-N demonstrated distinct reclassification patterns for malignant and benign lesions. Among the 92 pathologically malignant lesions, 15 lesions were upgraded on MammouS-N relative to hand-held ultrasound (HHUS), whereas 26 lesions were downgraded, resulting in a net downgrade difference of 11 lesions, corresponding to a net downgrade rate of 12.0%. The receiver operating characteristic (ROC) curve analysis yielded AUC = 0.893 (95% CI, 0.844–0.943) for MammouS-N and 0.923 (95% CI, 0.885–0.962) for HHUS, without significant difference (*p* = 0.207, DeLong’s test) (Fig. 3).

**Table 3:**
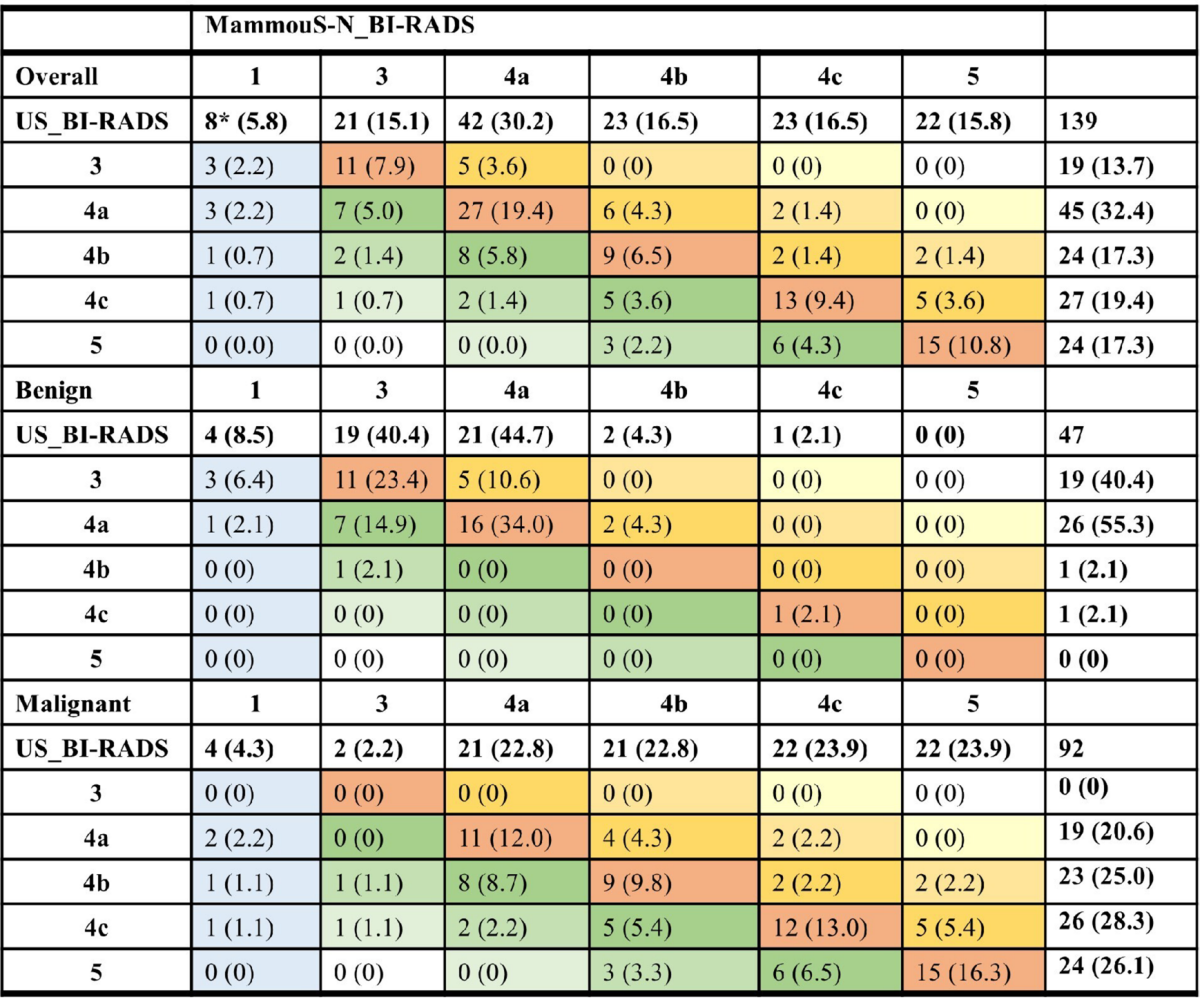
BI-RADS reclassification by MammouS-N compared with HHUS according to pathological diagnosis. Percentages are calculated based on the total number of benign (*n* = 47) or malignant (*n* = 92) lesions, respectively. Yellow indicates BI-RADS category upgrading by MammouS-N relative to HHUS; green indicates category downgrading; orange indicates concordant BI-RADS categorization; light blue indicates lesions not detected by MammouS-N (*Not detected 8 lesions were classified as BI-RADS category 1 for analysis).

### Factors influencing lesion detectability

**Fig. 4:**
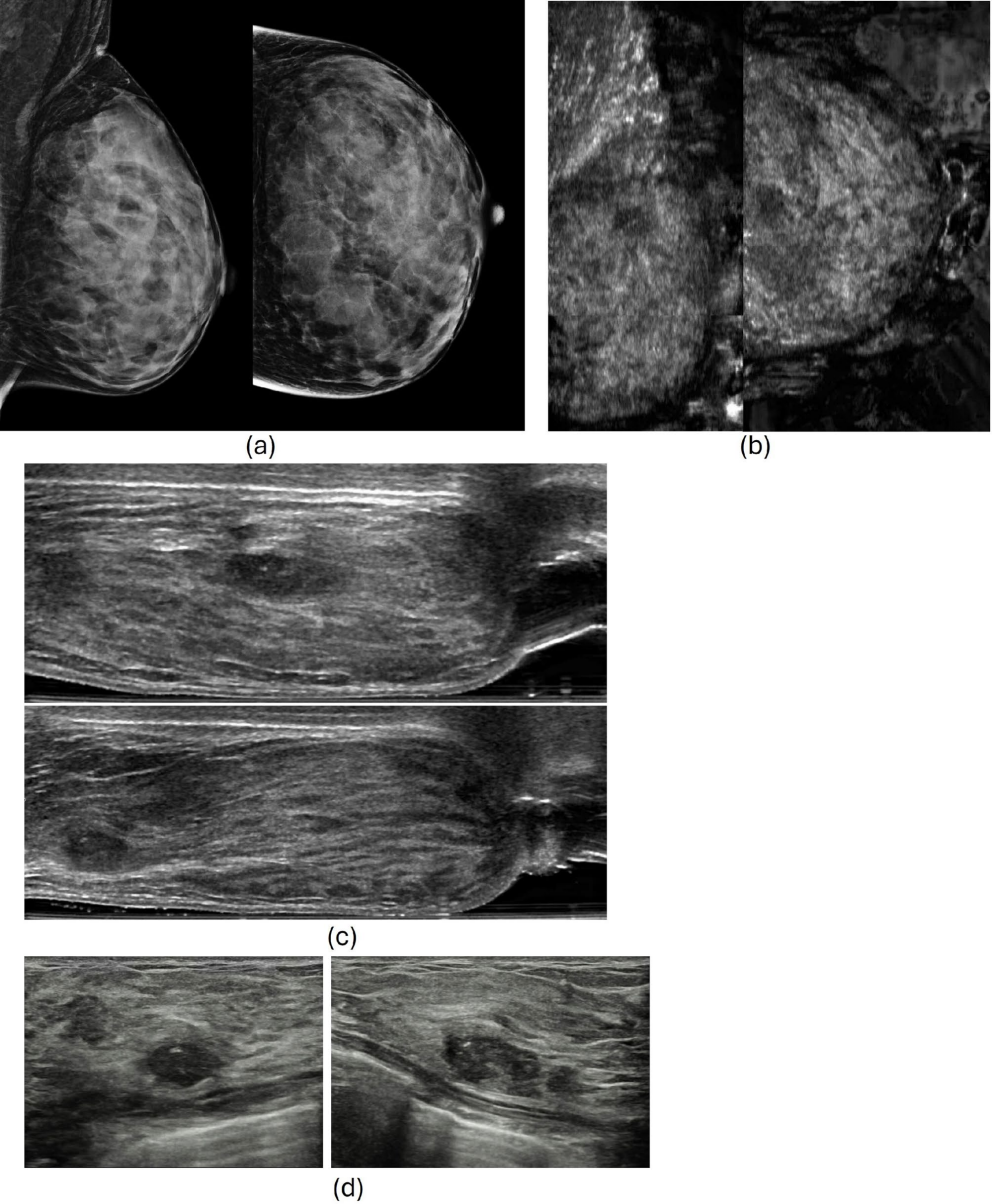
A 39-year-old woman with invasive ductal carcinoma (ER 90%, PR 40%, HER2 3+, Ki-67 83.6%). (**A**) Mammogram shows no distinct lesion due to extremely dense parenchyma (BI-RADS composition grade D), consistent with a mammographically occult cancer. (**B**) Reconstructed MLO (left) and CC (right) MammouS-N views clearly depict tumor margins and internal echotexture visualized on MammouS-N. (**C**) Mediolateral oblique (MLO) axial scan (above) and craniocaudal (CC) axial scan (below) on MammouS-N show irregular hypoechoic mass which is suspicious for malignancy. (**D**) Corresponding hand-held ultrasound image of the breast mass demonstrates similar morphologic features.

Lesion detectability on MammouS-N did not significantly differ according to image quality parameters, including resolution, anatomic differentiation, tissue contrast, lesion conspicuity, and artifact (all *p* > 0.05, Table 4). When image quality was dichotomized as comparable or higher (score ≥ 4) versus inferior (< 4), no parameter was significantly associated with detection. All lesions presenting as mass-type findings on mammography were detected on MammouS-N. In comparison, 91.4% (32 of 35) of lesions associated with microcalcifications and 86.9% (33 of 38) of occult lesions were detected on MammouS-N (*p* = 0.027). Although non-visualized (occult) lesions on mammography tended to have lower detection rates on MammouS-N, the difference did not reach statistical significance when binary grouping (visible vs. occult) was applied (*p* = 0.086, Fig. 4).

**Table 4:** Relationship between image variables and lesion detectability on MammouS-N.

		Detection		
	Level	0	1	p-value
n		8	131	
Imaging quality				
Artifact_nipple	< 4	1 (12.5)	28 (21.4)	0.880
	>=4	7 (87.5)	103 (78.6)	
Anatomy_differentiation	< 4	0 (0.0)	9 (6.9)	0.979
	>=4	8 (100.0)	122 (93.1)	
Resolution	< 4	0 (0.0)	8 (6.1)	> 0.99
	>=4	8 (100.0)	123 (93.9)	
Tissue_contrast	< 4	0 (NA)	9 (6.9)	NA
	>=4	0 (NA)	122 (93.1)	
Lesion_conspicuity	< 4	0 (NA)	9 (6.9)	NA
	>=4	0 (NA)	122 (93.1)	
Mammographic findings				
Mass		0 (0.0)	56 (46.3)	0.027
Calcification associated		3 (37.5)	32 (26.4)	
Not detected		5 (62.5)	33 (27.3)	

## Discussion

This study presents a prospective exploratory evaluation of the newly developed automated breast ultrasound system, MammouS-N and investigates its clinical feasibility and diagnostic performance. Despite image-quality limitations and acquisition-related artifacts affecting whole-breast visualization, lesion-level image quality was comparable to conventional HHUS in most cases, with an overall detection rate of 90%. These findings suggest that MammouS-N can serve as a reliable adjunct imaging tool in the detection and characterization of breast lesions.

We used a scoring system of imaging quality that could potentially affect diagnostic performance and provided an objective framework for consistent evaluation. With this system, MammouS-N achieved acceptable or equal ratings relative to HHUS in over 90% of cases, across all categories, except for artifacts (79.1%). Imaging artifacts were occasionally noted, mainly due to the inherent imaging geometry of the prototype and the small air bubbles trapped in the coupling medium. These technical factors led to minor local signal dropouts or streaks but did not significantly affect lesion detection in our study. The gel used in this examination was formulated and applied using a technique specifically designed to minimize air bubble formation and improve acoustic coupling. Further refinement of image acquisition protocols and post-processing algorithms is warranted to minimize such effects and ensure optimal image uniformity.

**Fig. 5:**
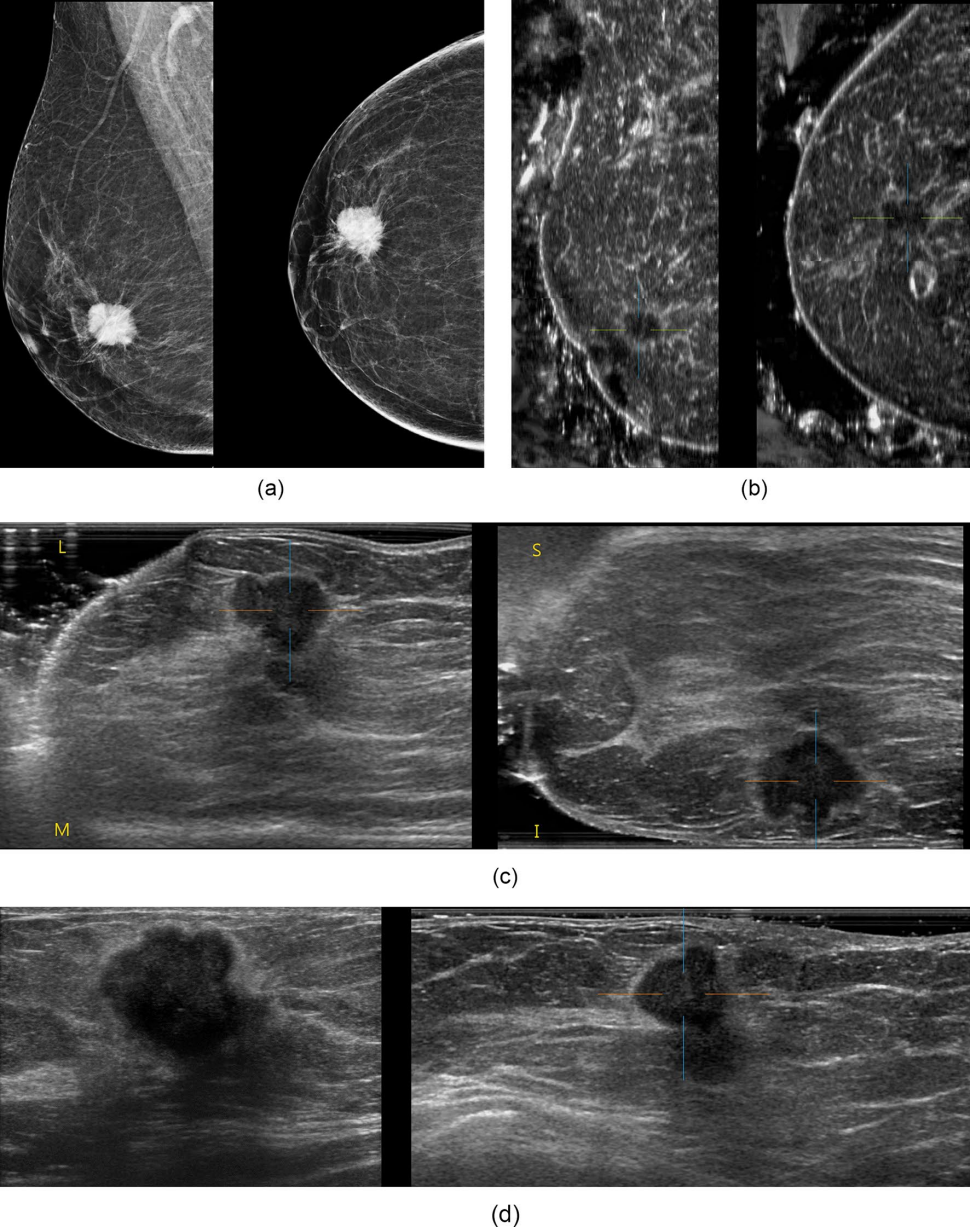
A 58-year-old woman with papillary carcinoma (ER 95% PR 70%, HER2 1+, Ki-67 21.4%). (**A**) Mammogram shows a mass in the right lower outer quadrant with scattered fibroglanduar (BI-RADS composition grade B). (**B**) MammouS-N reconstructed MLO (left) and CC (right) image demonstrates a corresponding hypoechoic lesion in the same projection, with clear spatial alignment to the mammographic finding. (**C**) MLO axial scan (left) and CC axial scan (right) on MammouS-N show irregular hypoechoic mass which is suspicious for malignancy. (**D**) Hand-held ultrasound (left) and PA view (right) confirm a concordant hypoechoic mass at the same location.

Assessment of coverage adequacy further confirmed the comprehensive volumetric imaging capability of MammouS-N. This evaluation provided an objective measure of the extent of imaging and verified that the majority of cases achieved full-breast visualization. The structured scoring allowed reproducible assessment of breast anatomy and lesion conspicuity, ensuring standardization across all imaging projections. These findings indicate that the MammouS-N system provided comprehensive volumetric imaging in the majority of cases, with the posteroanterior (PA) projection yielding the most consistent full-breast coverage. Of the 139 examinations, coverage adequacy was rated complete (score = 4 or 5) in 137 cases (98.6%), while approximately one-fourth of the CC view cases were classified as partial or limited coverage, reflecting technical constraints inherent to the current prototype design. Therefore, optimization is required to ensure full breast coverage, including the far-peripheral /deep posterior or upper medial breast, which are known blind spots also observed in conventional mammography. Adjusting the field of view (FOV) through additional projections, such as the PA view, could further enhance comprehensive volumetric coverage — similar to imaging techniques employed in other commercially available three-dimensional ultrasound systems^12^. However, the consistent imaging direction aligned with mammography is a unique advantage of this system, making it easier and more intuitive for radiologists to learn and adapt. This design enables intuitive spatial matching between mammographic and sonographic images, potentially enhancing diagnostic efficiency in multimodal imaging. A representative example demonstrating clear spatial correlation among mammography, MammouS-N, and hand-held ultrasound is shown in Fig. 5.

In clinical practice, this feature may be particularly useful in diagnostic workflows that require precise lesion localization and cross-modality correlation, such as problem-solving examinations or supplemental ultrasound following mammographic findings. By reducing the cognitive effort required to mentally translate lesion locations across different imaging planes, MammouS-N may facilitate more efficient and reproducible interpretation when correlated with digital mammography and digital tomosynthesis, particularly in the evaluation of non-mass lesions with or without associated microcalcifications. Furthermore, when used alongside contrast-enhanced mammography (CEM), MammouS-N could improve detection of the sonographic correlates of enhancing lesions. Ongoing optimization of probe design, coupling techniques, and reconstruction algorithms is expected to further improve image quality and diagnostic robustness in future iterations.

This study is the first to investigate and evaluate the clinical potential and diagnostic performance of MammouS-N. The overall lesion detectability of MammouS-N was high, with 131 of 139 lesions (94.2%) successfully visualized. The AUC for malignant lesion detection was 0.893 (95% CI, 0.844–0.943; *p* = 0.207), comparable to that of HHUS, indicating that the device rarely missed clinically significant cancers. A moderate to substantial intermodality agreement (Cohen’s κ = 0.453, weighted κ = 0.647) with a strong ordinal association (*p* < 0.001, Mantel–Haenszel χ² test) was observed, indicating consistent diagnostic alignment without systematic overestimation or underestimation of malignancy risk. To clarify the clinical implications of BI-RADS category shifts between MammouS-N and HHUS, reclassification patterns were analyzed separately according to pathologic outcome. Overall, MammouS-N demonstrated diagnostic alignment with HHUS, while showing a subtle tendency to assign slightly lower BI-RADS categories in certain malignant cases, possibly due to its compressed imaging geometry. This benign-leaning bias may initially lead to underestimation of lesion suspicion but also contribute to a reduction in false-positive rates, underscoring the importance of an interpretive learning curve.

The present study has several limitations. Although diagnostic performance metrics were compared between MammouS-N and hand-held ultrasound, this study was not designed as a formal non-inferiority or equivalence trial. Rather, it was conducted as an exploratory feasibility study to evaluate lesion detectability and diagnostic concordance in a clinical setting. First, it was conducted at a single institution with a limited number of patients, introducing potential selection and observer bias. Because the inclusion criteria favored women with known or suspected breast lesions, the prevalence of malignancy was higher than in the general screening population. Although image interpretation was performed in a blinded manner, diagnostic expectations could not be entirely excluded. Therefore, lesion detectability in population-based screening settings may be lower than that observed in this study. Future large-scale, multi-center trials are warranted to validate these findings in a broader cohort. Additionally, several lesions (*n* = 8, 5.7% of 139 lesions) were not detected by MammouS-N, likely due to restricted scan coverage or deep peripheral location beyond the imaging field. Expanding the scanning range and improving spatial resolution could further enhance the extent of the diagnosis and sensitivity of this device. Finally, although MammouS-N offers the advantage of acquiring ultrasound images in the same orientation as mammography, it is important to note that this system does not perform mammography or DBT simultaneously within a single device. Therefore, the potential benefits of acquiring both modalities in the same projection—particularly for second-look ultrasound targeting mammographically detected calcifications not visible on conventional ultrasound—remain underexplored. If a true hybrid device capable of integrating both imaging modalities were to be developed, such synergistic advantages could be better evaluated. Although earlier prototypes of such systems have been attempted, further research is warranted^13^.

This prospective study demonstrated the validity of a recent three-dimensional automated breast ultrasound (ABUS) prototype, MammouS-N, in a real-world clinical setting. The device effectively detected mammographically-positive or occult malignant breast lesions with high sensitivity and exhibited image quality comparable to that of conventional HHUS, supporting its potential as a complementary diagnostic tool. To further enhance its clinical utility, technical improvements in image quality optimization and scan coverage are warranted, along with the development of hybrid systems integrating MammouS-N with digital mammography. Future large-scale studies are needed to validate its diagnostic performance and to determine its potential role in population-based breast cancer screening, particularly for women with dense breasts.

## Supplementary Information

Below is the link to the electronic supplementary material.

Supplementary Material 1

## Data Availability

The raw data analyzed in the study are available from the corresponding author on reasonable request.
