# Supplementary material for: Evaluation of breast lesions using a newly developed 3D breast ultrasound imaging device: a preliminary study on efficacy and validity
Source: Sci Rep. 2026 Apr 27;16:19298. doi: 10.1038/s41598-026-48519-x (PMC13284351; doi:10.1038/s41598-026-48519-x)
Supplement: Supplementary file 1 — Supplementary Material 1 [file 41598_2026_48519_MOESM1_ESM.docx]

***Supplemental Table 1. Assessment of breast coverage according to MammouS-N projections***

| **Coverage** | **MLO per exam** | **CC per exam** | **PA per exam** | **Overall per breast** |
| --- | --- | --- | --- | --- |
| **5-complete** | **68 (49.3)** | **54 (41.5)** | **121 (87.7)** | **124 (89.2)** |
| **4-near-complete** | **60 (43.5)** | **46 (35.4)** | **13 (9.4)** | **13 (9.4)** |
| **3-partial** | **9 (6.5)** | **28 (21.5)** | **3 (2.2)** | **2 (1.4)** |
| **2-limited** | **1 (0.7)** | **2 (1.5)** | **1 (0.7)** | **0 (0)** |
| **1-inadequate** | **0 (0)** | **0 (0)** | **0 (0)** | **0 (0)** |
| **NA*** | **1** | **9** | **1** | **0** |

****Views excluded from analysis due to suboptimal image quality due to artifact or absence of exam in the corresponding projection (MLO, CC, or PA)***
